# Reappraisal of *Vipera aspis* Venom Neurotoxicity

**DOI:** 10.1371/journal.pone.0001194

**Published:** 2007-11-21

**Authors:** Elisabeth Ferquel, Luc de Haro, Virginie Jan, Isabelle Guillemin, Sabine Jourdain, Alexandre Teynié, Jacques d'Alayer, Valérie Choumet

**Affiliations:** 1 Unité des Venins, Institut Pasteur, Paris, France; 2 Centre Antipoison, Hôpital Salvator, Marseille, France; 3 Ciphergen, Paris, France; 4 UENC INRA, Centre de Recherche de Theix, Saint Genes Champanelle, France; 5 Plate-forme d'Analyse et de Microséquençage des Proteines, Institut Pasteur, Paris, France; Muséum National d'Histoire Naturelle, France

## Abstract

**Background:**

The variation of venom composition with geography is an important aspect of intraspecific variability in the *Vipera* genus, although causes of this variability remain unclear. The diversity of snake venom is important both for our understanding of venomous snake evolution and for the preparation of relevant antivenoms to treat envenomations. A geographic intraspecific variation in snake venom composition was recently reported for *Vipera aspis aspis* venom in France. Since 1992, cases of human envenomation after *Vipera aspis aspis* bites in south-east France involving unexpected neurological signs were regularly reported. The presence of genes encoding PLA_2_ neurotoxins in the *Vaa* snake genome led us to investigate any neurological symptom associated with snake bites in other regions of France and in neighboring countries. In parallel, we used several approaches to characterize the venom PLA_2_ composition of the snakes captured in the same areas.

**Methodology/Principal Findings:**

We conducted an epidemiological survey of snake bites in various regions of France. In parallel, we carried out the analysis of the genes and the transcripts encoding venom PLA_2_s. We used SELDI technology to study the diversity of PLA_2_ in various venom samples. Neurological signs (mainly cranial nerve disturbances) were reported after snake bites in three regions of France: Languedoc-Roussillon, Midi-Pyrénées and Provence-Alpes-Côte d'Azur. Genomes of *Vipera aspis* snakes from south-east France were shown to contain ammodytoxin isoforms never described in the genome of *Vipera aspis* from other French regions. Surprisingly, transcripts encoding venom neurotoxic PLA_2_s were found in snakes of Massif Central region. Accordingly, SELDI analysis of PLA_2_ venom composition confirmed the existence of population of neurotoxic *Vipera aspis* snakes in the west part of the Massif Central mountains.

**Conclusions/Significance:**

The association of epidemiological studies to genetic, biochemical and immunochemical analyses of snake venoms allowed a good evaluation of the potential neurotoxicity of snake bites. A correlation was found between the expression of neurological symptoms in humans and the intensity of the cross-reaction of venoms with anti-ammodytoxin antibodies, which is correlated with the level of neurotoxin (vaspin and/or ammodytoxin) expression in the venom. The origin of the two recently identified neurotoxic snake populations is discussed according to venom PLA_2_ genome and transcriptome data.

## Introduction

Snake venoms are complex mixtures of biologically active proteins. They contain several enzymes and toxins that act in synergy to fulfill the two main functions of the venom that are subduing and digesting prey. Diversity of snake venom within species has repeatedly been described over the last twenty years. The variation of venom composition with geography is an important aspect of this intraspecific variability. Several species of medical importance belonging to *Viperidae* and *Elapidae* families produce different clinical symptoms across the geographical range of their distribution [1], [2], [3], [4], [5]. The causes of this variability remain unclear [6], [7]. In some cases the validity of the definition of the species is necessary and the apparent intraspecies variation is really interspecies variation. Interspecies hybridization may be a major mechanism of diversification of the composition of snake venoms. Whatever the origin, the diversity of snake venom is important both for our understanding of venomous snake evolution and for the preparation of relevant antivenoms to treat envenomations [5], [6], [8], [9].

**Figure 1:**
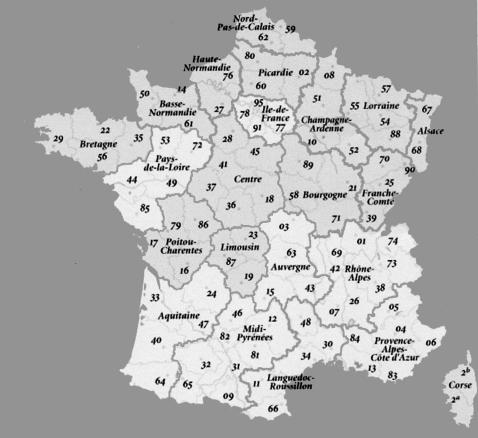
Regions of France involved in the medical and herpetological survey of *Vipera aspis* envenomation. The regions and departments (indicated by numbers) where the medical and herpetological surveys were conducted are shown in light grey.

Three species of venomous snakes are of medical importance in Europe: *Vipera aspis*, *Vipera berus* and *Vipera ammodytes* (*Vam*). The clinical features of *Vipera aspis aspis* (*Vaa*) and *Vipera berus berus* (*Vbb*) envenomations are mostly local, but can be associated with systemic signs (gastrointestinal and coagulation disorders, low blood pressure) in cases of severe envenomation [10], [11]. Envenomation by *Vipera ammodytes ammodytes (Vamam)*, a species located in Eastern Europe, can cause neurological symptoms [12]. *Vamam* venom contains neurotoxic presynaptic phospholipases A_2_ (PLA_2_), the three isoforms of single chain ammodytoxin (AtxA, AtxB, AtxC) [13], whereas the venom of *Vipera ammodytes meridionalis* (*Vammer*), another *Vam* subspecies of northern Europe, contains vipoxin, an heterodimeric postsynaptic PLA_2_ neurotoxin [14], [15], [16]. A geographic intraspecific variation in snake venom composition was recently reported for *Vaa* venom in France. Since 1992, the Marseille Poison Center has regularly observed cases of human envenomation after *Vaa* bites in south-east France involving unexpected neurological signs [17], [18]. In France, neurological symptoms had previously only been described in cases of envenomation by a subspecies of *Vipera aspis*, *Vipera aspis zinnikeri* (*Vaz*) located mainly in the south-west of France (Aquitaine, Midi-Pyrénées, see Figure 1) [19]. *Vaz* venom neurotoxicity is due to a postsynaptic neurotoxin, PLA_2_-I, homologous to vipoxin [20].

The viper responsible for one of these “neurotoxic” envenomations was classified as *Vaa* on the basis of its morphology [17]. The venom gland mRNA of this neurotoxic *Vaa* snake was sequenced and led to the identification of two neurotoxins, AtxB, an isoform of ammodytoxin previously described only in the venom of *Vamam*, and vaspin, a neurotoxin similar to PLA2-I and vipoxin [21], composed of two subunits, an acidic one called vaspin A (vasA) and a basic one, called vaspin B (vasB). Among the genes encoding the venom PLA_2_s of the same neurotoxic snake were genes encoding ammodytin I1 (AmI1), ammodytin I2 (AmI2), vaspin and the three isoforms of Atx, as well as a retrotransposon reported to be a phylogenetic marker of the genome of *Vipera ammodytes* species [22], [23]; a true *Vaa* genome only contains genes encoding AmI1, AmI2 and vaspin from this list. These observations indicate that there is a previously undescribed population of *Vaa* snakes in the south-east of France. This led us to investigate any neurological symptoms associated with snake bites in other regions of France and in neighboring Switzerland and Italy. We also conducted a biochemical and an immunological study of *Vipera* snake venoms in the same regions to evaluate the potential neurotoxicity of the venom samples. In parallel, we analyzed the venom PLA_2_ composition of the genome and of the transcriptome of some snakes. The results allow a better definition of *V. aspis* sp. venom neurotoxicity. Analysis of the cross-reactivity of the venoms with anti-ammodytoxin antibodies indicated that only a restricted population of *Vaz* snakes found only in areas of contact with other snake species is able to cause neurological symptoms. We also describe a geographical variation in the composition of *Vaa* venom in the southwestern part of “Puy-de-Dôme” department: it is indistinguishable from a pure *Vaz* venom.

## Materials and Methods

### Material

Pooled *Vaa* and pooled *Vaz* venoms were purchased from Latoxan (Valence, France).

### Methods

#### Epidemiological and clinical analysis of snake bites and ecological analysis of *Vipera* snakes

This study is not a clinical trial. The patients were treated as usual like every envenomed victims in France, explaining why no special approval was necessary. The sole difference was the clinical feature with a new observed neurotoxicity, but there were no treatment protocol modifications in the medical management. Analytical assays were performed in blood samples collected for the classical exploration of bitten patients in order to evaluate the clinical gradation. Snake bites were analyzed retrospectively in 7 regions of France (Aquitaine, Auvergne, Midi-Pyrénées, Languedoc-Roussillon, Pays de Loire, Provence-Alpes-Côte d'Azur, Rhone-Alpes) from 1990 to 2002 (Figure 1). Unusual cases of neurotoxic envenomation reported by the Marseille Poison Centre from 2003 to 2005 were also included. Patient data (age, sex, time of bite, clinical manifestations and treatment) were collected. The severity of envenomation of each patient was determined according to the classification established by [24]. Grade 1 envenomations (mild envenomations) were identified by the presence of local signs and absence of systemic symptoms; grade 2 (moderate envenomations) by regional oedema and moderate systemic symptoms; grade 3 (severe envenomation) by extensive oedema and severe systemic symptoms. Epidemiological data from 110 patients that presented a minor, moderate or severe envenomation were collated using Excel and Epi-Info software. We then excluded from the study cases of envenomation for which no indication of department was mentioned.

Authorization for capture and transportation of *Vipera aspis* and *Vipera berus* snakes in France were obtained from the prefectures of the following French administrative departments: “Alpes de Haute-Provence” (code no. 04), “Alpes-Maritimes” (code no. 06), “Puy-de-Dôme” (code no. 63), “Ariège” (code no. 09), “Haute-Pyrénées” (code no. 65), “Gironde” (code no. 33), “Hérault” (code no. 34), “Loire-Atlantique” (code no. 44), “Haute-Savoie” (code no. 74), and “Seine-et-Marne” (code no. 77) (see Figure 1). For *Vipera ursinii* (*Vu*) and *Vam* capture, transportation authorizations were obtained from the Ministry of Ecology and Sustainable Development of France. *Vipera ammodytes montandoni* (*Vammon*) snakes were captured at Strazha (Bulgaria), *Vipera aspis francisciredi* (*Vaf*) in Piemont (Italy), *Vipera aspis huguy* (*Vah*) in Calabria (Italy) and *Vipera latastei latastei* (*Vll*) in the region of Burgos (Spain). Seventy-two snakes were collected. The scientists in charge of snake captured in the various regions filled in a questionnaire. The data collected, indicating the sex, age and biotope (precise geographical localization, altitude) for each animal captured in France, are listed in Supplementary Table S1. They were identified by use of classical keys (including the numbers of ventral and sub ocular plates, scales between the labial plates and the eyes). When the dorsal pattern was clearly distinguishable from that of other *Vipera aspis* snakes from the same department, the subspecies was not indicated and replaced by a question mark. Snakes were milked, then killed and their organs were kept at −80°C until analysis. Additional *Va* snake venoms were collected in the south-west of France and *Vaf* snake venoms were collected in three regions of Italy: Calabria, Liguria and Tuscany.

#### PLA_2_ transcript preparation from venomous gland and genomic DNA extraction

Total RNA was extracted as described previously [25]. RT-PCR, ligation, molecular cloning, plasmid DNA extraction and sequencing reactions were performed as previously described [26]. Total genomic DNA from tissue samples (liver, muscles, kidney or skin) was extracted as described by [27].

#### PLA_2_ gene screening by PCR

PCR was performed using genomic DNA and/or cDNA extracts. The primers specific to each PLA_2_ species were designed to confirm whether a given type of transcript was present in the venom gland of a specimen, or whether the corresponding gene was present in the genome of the specimen. AmI1 and AmI2 were amplified with 5′gacggggatatttggtataatgtcc3′/3′gcaatgagaggatgggtagttc5′ and 5′gggaacctttaccagttcggg3′/3′gcaatgagaggatgggtagttc5′, respectively. The acidic subunits of vaspin and vipoxin were amplified with 5′caaaagacggggaaagaagc3′/3′tcctccgtgcaatgagagattg5′ primers. The basic ones were amplified with 5′gcctgctcgaattcgggatg3′/3′gtctgcctgcatctagagga5′. The Bov-B line retroposon was amplified with 5′gagtggatgcacagtcgttg3′/3′ctccttcttgcacaaaaagtg5′ primers. Ammodytin L (AmL) was amplified using the following set of primers: 5′ gtgatcgaatttgggaag atgatcca3′/3′cccttgcatttaaacctcaggtacac5′. The primers for AtxB PLA_2_ were 5′gcctgctcgaattcgggatg3′/3′ctccttcttgcacaaaaagtg5′ whereas, 5′ctgctcgaattcgggatg3′/3′ gtcygggtaattcctatata5′ were specific for AtxA or C. The PCR products obtained with primers specific to Atx A/C were purified and incubated with restriction enzyme *Hpa*II for 4 hours at 37°C to test for the presence of AtxA. AtxA contains an extra *Hpa*II site to AtxC, yielding four cDNA fragments (63, 51, 129 and 201 bp) instead of three (63, 180 and 201 bp).

#### Determination of protein concentration

the protein concentration of venom samples was determined using the Coomassie plus method (Pierce, Rockford, Il, USA).

#### Fractionation of pooled *Vaa* and pooled *Vaz* snake venoms

The fractionation of pooled *Vaa* and pooled *Vaz* venoms was performed on a column containing Sephadex G100 superfine and using ammonium formiate 1M, pH 3.5 as running buffer. Fractions of 450 µl were collected at a flow rate of 2.6 ml per hour. The protein concentration of each fraction was measured on a spectrophotometer at the wavelength of 280 nm. Fractions corresponding to the same peak were pooled and tested for their PLA_2_ activity by fluorimetry [28].

#### Microsequencing

Venoms 406 and 508 were subjected to PAGE (20% SDS-polyacrylamide gel) in reducing conditions. For venom 406, the gel was rinced three times with water and stained overnight with Amidoblack. A piece of gel containing Atx PLA_2_ was cut out and digested with 2 µg/ml of endoproteinase Lys-C (Boehringer Mannheim GmBH, Mannheim, Germany) for 18 h at 35°C. The digested material was injected onto a DEAE column linked to a C-18 reversed-phase HPLC column and eluted with a 2–70% acetonitrile gradient in 0.1% trifluoroacetic acid. The molecular weight of eluted pepides was determined by surface enhanced laser desorption ionization-time of flight-mass spectrometry (SELDI-TOF-MS) on a Au proteinChip and the peptide corresponding to the C-terminal part of Atx was sequenced on a protein sequencer (494; Applied Biosystems, Foster City, CA). For venom 508, after the end of the electrophoresis, the gel was transferred onto PVDF overnight at a constant voltage of 15 V. The PVDF membrane was then stained with Amidoblack and the band containing low molecular weight PLA_2_s was excised and subjected to Edman degradation on an Applied Biosystems 494 sequencer.

#### Analysis of snake venom PLA_2_ contents by SELDI-TOF-MS

Venoms were diluted to 1.25 mg/ml in distilled water and spotted onto a variety of chemical surfaces: reverse phase H4 or H50 (hydrophobic surface: C-16 or C-4 (long chain aliphatic), weak cation exchanger (WCX2: carboxylate) or strong anion exchanger (SAX2: quaternary ammonium). Best and similar profiles were obtained with WCX2 and H4. H4 chips were activated with 10% acetonitrile and air dried prior to sample incubation. Venom samples (2.5 µg, final volume: 2 µl) were spotted, allowed to dry and spots were washed four times with 5 µl of distilled water. The samples were then air dried. WCX2 chips were equilibrated twice for 10 min with 150 µl of 100 mM sodium acetate pH 3.8, using a bioprocessor. Venom samples (2.5 µg) were diluted in 100 µl of 100 mM sodium acetate pH 3.8 containing 0.1% Triton and incubated on the chips for 30 min at room temperature under shaking. Then, the chips were washed: first for 5 min with 100 µl of 100 mM sodium acetate pH 3.8 containing 0.1% Triton, second, twice with 100 µl of 150 µl of 100 mM sodium acetate pH 3.8 and finally twice with double-distilled H_2_O. The samples were then air dried. Then, 0.5 µl of sinapinic acid (Ciphergen) saturated in 0.5 µl of 50% acetonitrile–0.5% trifluoroacetic acid was applied twice on each spot (WCX2 or H4 ProteinChips) and the spots were air dried. Molecules retained on the surfaces were visualized by reading the spots of each array in a SELDI-TOF-MS reader (PBSII; Ciphergen). Spectra were generated with 175 shots averaged at 210 laser intensity with a detector sensitivity of 7 and an accelerating voltage of 20 kV in positive mode with automatic data collection software 3.0 program. External mass calibration curve was performed on one spot of each array by using ubiquitin (8564.8 Da), cytochrome C (12230.9 Da) and beta-lactoglobulin A (18363.3 Da).

Raw spectra were processed and analysed with the Ciphergen Express data manager software version 3.0 (CE; Ciphergen Biosystems). Spectra were externally calibrated with ubiquitin (bovine) (8564.8+1H), cytochrome C (bovine) (12230.9+1H), and beta-lactoglobulin A (bovine) (18363.3+1H). The baseline was established using default parameters and spectral intensities were normalized by total ion current (TIC). Consistent peak sets of similar mass across the spectra were generated with Ciphergen Express Cluster Wizard. This Wizard operates in three passes across the spectra. The first pass performs peak detection at high signal-to-noise ratio (s/n) to pick out well-defined peaks as starting points for forming clusters. A second pass selects lower s/n ratio peaks, within a mass window defined around the first pass peaks. The algorithm completes the clusters in a third pass by creating artificial peaks where none were detected in the first two passes, at the exact centre of clusters. In this analysis, the first pass was performed with an s/n threshold of 5, and the second pass with an s/n threshold of 2, in a 0.2% width mass window. Estimated peaks were added to complete clusters. Clusters were assembled between 13000 and 14000 Da, the expected size of venom toxins. The cluster lists contained normalized peak intensity values for each sample within a group and P-values were calculated between the medians of the peak intensities to detect significant differences in abundance for particular proteins.

#### Preparation of anti-ammodytoxin antibodies

Rabbit polyclonal antibodies against AtxA (Latoxan, Rosans, France) were prepared as described by [29].

#### Determination of venom immunological crossreactions by ELISA

We determined by western blot the cross-reactivity of anti-AtxA antibodies (data not shown). They react with vasB and Atx but don't bind AmI1 or AmI2. Microtitration plates (Nunc, Roskilde, Denmark) were coated by incubation overnight at 4°C or 1 hr at 37°C with 100 µl of venom solutions (5 µg/ml) diluted in carbonate buffer (0.01M, pH 9.5). The plates were washed and incubated for 1 hr at 37°C with 100 µl of various dilutions of rabbit polyclonal anti-AtxA antibodies in PBS containing 3% BSA (PBS-BSA), then washed again. Peroxidase-labeled anti-rabbit IgG (100 µl of 1/1000 dilution in PBS-BSA) (Cappel/ICN Biomedicals, Aurora, Ohio, USA) were added to the wells and the plates were incubated for 1 hr at 37°C. After washing, substrate medium (100 µl of 10 mM sodium phosphate, pH 7.3, containing 2 mg/ml of OPD and 0.06% of perhydrol) was added to each well and the plates were incubated for 7 min in the dark at room temperature. The reaction was stopped by adding 50 µl of 0.5% sodium sulfite in 2N sulfuric acid to each well. Absorbance was measured at 490 nm using a microtitration plate reader (MR5000, Dynatech, France). Optical densities measured at dilutions of anti-AtxA IgG of 1/16,000 were used for cross-reactivity gradation: level 0: OD≤0.1, level 1: 0.1<OD≤0.2; level 2: 0.2<OD≤0.3; level 3: 0.3<OD≤0.4; level 4: OD>0.4.

## Results

### Epidemiological study of envenomations in France: detailed symptomatology of neurotoxic envenomations according to geographical region

**Figure 2:**
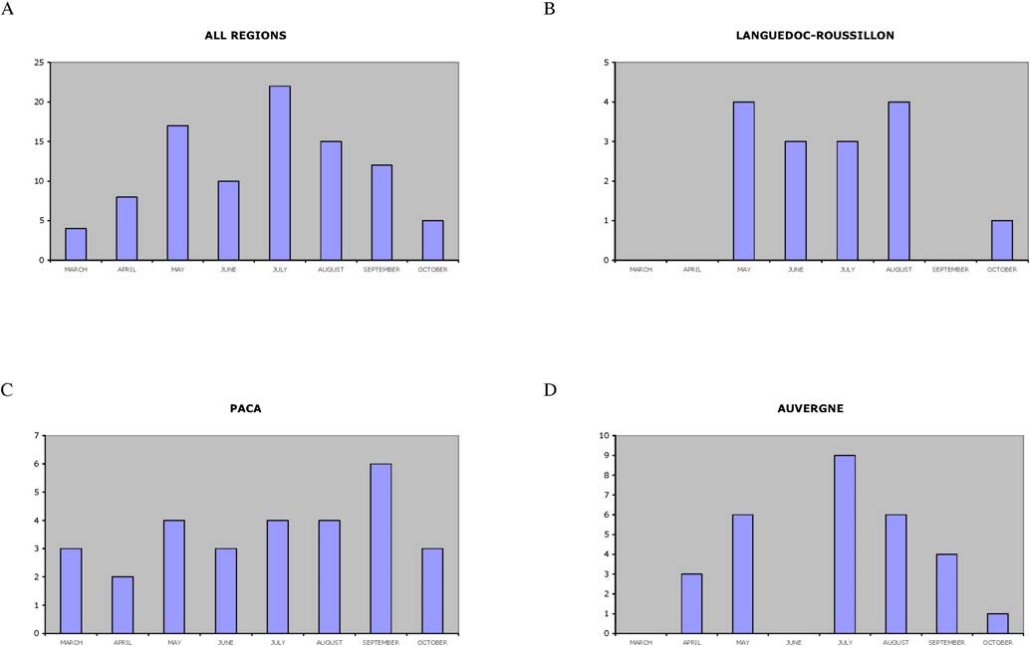
Annual distribution of envenomation cases in France.

**Figure 3:**
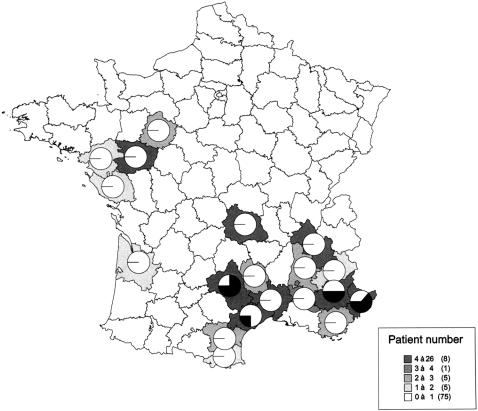
Distribution of classical and neurotoxic cases of envenomation in various regions of France. Pie charts show the relative numbers of classical (white) and neurotoxic (black) grade 2 and 3 envenomations. The total number of cases reported in each department is presented as grey levels, as indicated in the right bottom end corner of the figure.

A hundred and ten cases of envenomation were reported between 1990 and 2005 in various regions of France (Aquitaine, Auvergne, Midi-Pyrénées, Languedoc-Roussillon, Pays-de-Loire, Provence-Alpes-Côte d'Azur, Rhone-Alpes). Thirteen cases of envenomation for which no indication of department was mentioned were then excluded from the study and ninety-seven cases are reported in Supplementary Table S2. Envenomation cases were recorded from March to October (Figure 2A). They were distributed evenly from May to August in Languedoc-Roussillon (Figure 2B) and from March to October in Provence-Alpes-Côte d'Azur (PACA) (Figure 2C) whereas in Auvergne, peaks of envenomation were observed in May, July and August (Figure 2D). The age distribution was from 3 to 91 years. The severity of envenomation of each patient was determined according to the classification established by [24]. Fifteen cases of grade 1, 58 of grade 2 and 24 of grade 3 were reported. Neurological symptoms were only observed in grades 2 and 3 and constituted 17% of all moderate and severe envenomation cases. They were reported from April to October in four departments, “Alpes de Haute-Provence” (code no. 04), “Alpes-Maritimes” (code no. 06), “Aveyron” (code no. 12) and “Hérault” (code no. 34) (Supplementary Table S2). Figure 3 shows the prevalence of neurological symptoms in grade 2 and 3 envenomations for each department. The neurological signs are detailed in Table 1. They were mainly cranial nerve disturbances (ptosis, opthalmoplegia, paresthesia, diploplia and dysphagia). Ptosis was observed in 100% of the cases. Opthalmoplegia, drowsiness and dysphagia were recorded in all four departments. The most varied clinical symptoms were observed in “Alpes-Maritimes” and “Aveyron” with 9 different neurological signs (Table 1). We also analyzed the kinetics of appearance of each symptom. Drowsiness and diploplia were the most precocious symptoms, with mean appearance times of 5.83±2.63 hrs, 4.75±2.96 hrs after the envenomation, respectively. Ptosis and ophtalmoplegia were delayed, occurring after 9.03±5.6 hrs and 10±6.12 hrs.

**Table 1:** Clinical cases of neurotoxic envenomations

French departement	Alpes-de-Haute-Provence (04)	Alpes-Maritimes (06)	Aveyron (12)	Hérault (34)	Total
Case number	3	7	3	1	14
M/F	2/1	5/2	2/1	Not mentionned	9/4
Age in years (mini/maxi)	48/88	32/68	4/62	5	4/88
Years	1995 to 1999	1992 to 2005	1998 to 2004	2001	1992 to 2005
Grade 1/2/3 number	0/2/1	0/6/1	0/3/0	0/1/0	0/12/2
Local signs					
Local swelling	1	3	2	1	7
Extensive swelling	2	4	1	0	7
Neurological signs					
Ptosis	3	7	3	1	14 (100%)
Ophtalmoplegia	2	5	2	1	10 (71%)
Drowsiness	1	4	2	1	9 (64%)
Dysphagia	2	3	1	1	7 (50%)
Dysphonia	0	4	1	1	6 (43%)
Vision troubles *	1	3	1	0	5 (36%)
Paresthesia	0	3	1	0	4 (29%)
Muscle weakness	0	1	1	0	4 (14%)
Lip paralysis	0	2	2	0	4 (28%)
Agueusia	0	1	0	0	1 (7%)
Other systemic symptoms	3	7	3	0	13 (93%)
Antivenom treatment	3	4	2	1	10 (71%)

* accomodation trouble, diplopia

### Venom PLA_2_ screening at the genome and transcriptome level

To correlate the neurological signs observed with the venom composition, snakes were captured in the geographical regions in which the epidemiological analyses were conducted. Neurotoxins previously identified in the venoms of “neurotoxic” French vipers were phospholipases A_2_ or their homologs of 13 to 14 kDa. At least one snake of each region was sacrificed. cDNAs and genes encoding venom PLA_2_s were amplified from the venomous glands of *Vipera* snakes and probes specific of european viper venom PLA_2_s (AmI1, AmI2, vasA, vasB, AtxA/C and AtxB, AmL) were used for PLA_2_ profiling.

With regard to the French snake population, four classes can be identified based on their PLA_2_ genome composition (Table 2). The first class is characterized by the presence of genes encoding AmI1 and AmI2 and is only composed of *Vaatra* snake 651 and *Vaa* snake 564. Interestingly, this latter snake belongs to an isolated population of *Vaa* snake whose biotope is composed of garrigues and unlike other “classical” *Vaa* snakes has lost vaspin encoding genes. The second class, characterized by genes encoding AmI1, AmI2, vasA and vasB, is composed of *Vaa* snakes 303 (Loire-Atlantique), 452, 601, 502 (Puy-de-Dôme) and *V. ursinii* (*Vu*) snake 620 (Vaucluse). Although they differ in their genome composition, classes 1 and 2 have in common the unique expression of AmI1 and AmI2 in their venoms. The third class is composed of *Vaz* snakes 613 (Gironde) and 508 (Hautes-Pyrénées) and *Vaa* 456 (Puy-de-Dôme) and possesses genes encoding AmI1 and vaspin. All the snakes of this class present the same transcriptome profile for which all the venom PLA_2_ genes of the genome are expressed. The fourth class is composed of *Vipera aspis* snakes 406, 513, 514 (Alpes-de-Haute-Provence) and 603 (Alpes-Maritimes) and possesses genes encoding AmI1, AmI2, vasA, vasB, AtxA, AtxB and AtxC as well as the Bov-B line retroposon. Interestingly, their transcriptome profiles differ at the level of vaspin and ammodytoxin isoforms expression, viper 603 being the only one that expressed all neurotoxins genes. This viper was captured after having bitten a human, who thereafter suffered from ptosis, ophtalmoplegia, agueusia, paresthesia and muscle weakness [17], [18]. Snake 459 is the only *Vaz* specimen, which expressed AmI2 in its venom at the difference of *Vaz* 613 and 508 snakes that did not possess AmI2 gene in their genome (Table 2).

**Table 2:** Genome and transcriptome analysis of snake venom PLA_2_

Label number	Administrative French department or country	Viper	Genome composition									Venomous gland composition (cDNA)							
			AmI1	AmI2	vasA	vasB	AtxA	AtxB	AtxC	AmL	ART	AmI1	AmI2	vasA	vasB	AtxA	AtxB	AtxC	AmL
406	Alpes-de-Haute-Provence (04)	*Vaa*	yes	yes	yes	yes	yes	yes	yes	no	yes	yes	yes	no	no	yes	yes	yes	nd
513	Alpes-de-Haute-Provence (04)	*Va?*	yes	yes	yes	yes	yes	yes	yes	no	yes	yes	yes	nd	nd	nd	nd	nd	nd
514	Alpes-de-Haute-Provence (04)	*Va?*	yes	yes	yes	yes	yes	yes	yes	no	nd	yes	yes	no	no	no	yes	no	nd
603	Alpes-Maritimes (06)	*Vaa*	yes	yes	yes	yes	yes	yes	yes	no	yes	yes	yes	yes	yes	yes	yes	yes	nd
459	Haute-Garonne (31)	*Vaz*	nd	nd	nd	nd	nd	nd	nd	nd	nd	yes	yes	yes	yes	no	no	no	nd
564	Hérault (34)	*Vaa*	yes	yes	no	no	no	no	no	no	no	nd	nd	nd	nd	nd	nd	nd	nd
613	Gironde (33)	*Vaz*	yes	no	yes	yes	no	no	no	no	no	yes	no	yes	yes	no	no	no	nd
303	Loire-Atlantique (44)	*Vaa*	yes	yes	yes	yes	no	no	no	no	no	yes	yes	no	no	no	no	no	nd
452	Puy-de-Dôme (63)	*Vaa*	yes	yes	yes	yes	no	no	no	no	no	yes	yes	no	no	no	no	no	nd
502	Puy-de-Dôme (63)	*Vaa*	yes	yes	yes	yes	no	no	no	no	no	yes	yes	no	no	no	no	no	nd
601	Puy-de-Dôme (63)	*Vaa*	yes	yes	yes	yes	no	no	no	no	no	yes	yes	no	no	no	no	no	nd
456	Puy-de-Dôme (63)	*Vaa*	yes	no	yes	yes	no	no	no	no	no	yes	no	yes	yes	no	no	no	nd
508	Hautes-Pyrénées (65)	*Vaz*	yes	no	yes	yes	no	no	no	no	no	yes	no	yes	yes	no	no	no	nd
651	Haute-Savoie (74)	*Vaatra*	yes	yes	no	no	no	no	no	no	no	yes	yes	no	no	no	no	no	nd
620	Vaucluse (84)	*Vu*	yes	yes	yes	yes	no	no	no	no	no	yes	yes	no	no	no	no	no	nd
605	Italy	*Vaf*	yes	yes	yes	yes	no	no	no	no	no	nd	nd	nd	nd	nd	nd	nd	nd
604	Italy	*Vah*	yes	yes	no	no	nd	yes	nd	yes	yes	nd	nd	nd	nd	nd	nd	nd	nd
608	Spain	*Vll*	yes	yes	no	no	no	no	no	no	no	nd	nd	nd	nd	nd	nd	nd	nd
709	Bulgary	*Vammon*	yes	yes	no	no	nd	yes	nd	yes	yes	yes	yes	no	no	no	no	no	yes

Venom PLA_2_ encoding genes were amplified by PCR using specific probes from liver DNA (genomics) and venomous gland cDNA (transcriptome) as described in the Material and Methods section. ART: Bov-b line retroposon. nd: not determined.

We also analyzed the PLA_2_ genome composition of European snakes. The genome of *Vaf* snake captured in Piemont is similar to that of *Vaa* whereas that of *Vah* from Italy contains Atx and AmL but not vaspin, as does that of *Vammon* of Bulgaria. *Vipera latastei latastei* contains only AmI1 and AmI2 as *Vaatra* 651 and *Vaa* 564.

In addition to the complexity of the transcriptome profiles, the sequencing of transcripts encoding PLA_2_ genes in *Vaa* and *Vaz* snake venom glands has shown that three isoforms of AmI1 and two of AmI2 are expressed in the venom of these snakes: the molecular weight of AmI1 isoforms ranges between 13676 and 13694 Da and that of AmI2 isoforms between 13526 and 13553 Da [30]. Two isoforms of vasA were identified with molecular weights of 13655 and 13665 Da whereas only one isoform of vasB was characterized (13841 Da) [30]. All these molecular weights are indicated assuming that the 14 cysteine residues are engaged in disulphide bonds.

### PLA_2_ proteome of snake venoms from various French regions using SELDI-TOF-MS proteinchip

**Figure 4:**
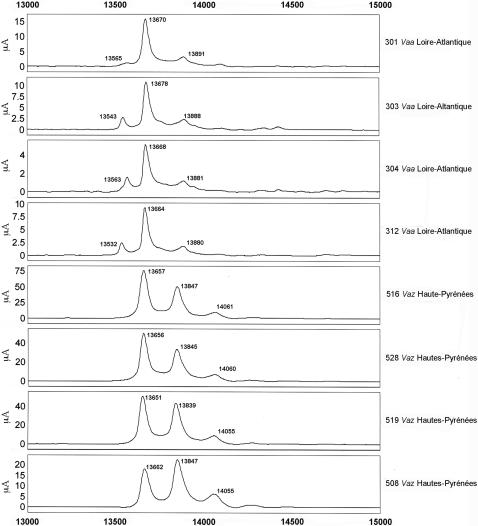
Selective capture of PLA_2 _using SELDI ProteinChip Array. Individual venoms (2.5 µg) were applied to the surface of a WCX2 ProteinChip as described in the Methods section. Unbound proteins and interfering substances were washed away and matrix was added and allowed to dry. The captured proteins were detected using surface enhanced laser desorption/ionization (SELDI) time-of-flight mass spectrometry. Normalised mass (m/z) for each peak (in Daltons (Da)) is demonstrated on the X-axis, while intensity (μA) is plotted on the Y-axis. m/z ratios from different spectra that are within 0.2% of each other are considered as potentially the same protein.

**Figure 5:**
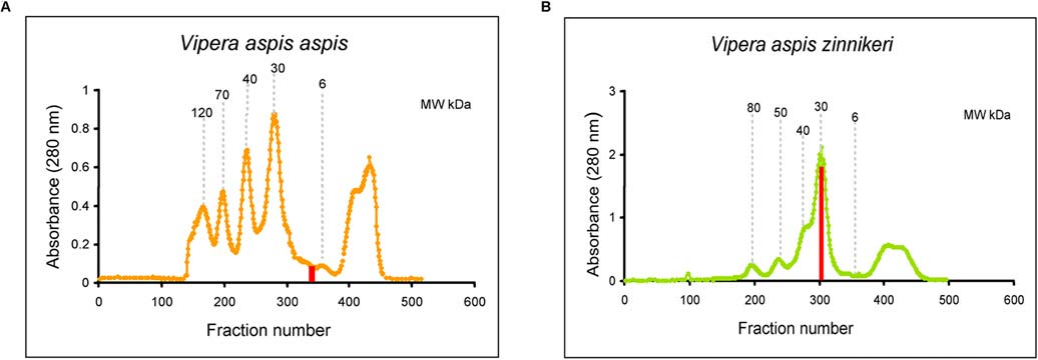
Isolation of PLA_2_ fractions from pooled *Vaa* and pooled *Vaz* venoms on Sephadex G100 Superfine column. (A) Pooled *Vaa* and (B) pooled *Vaz* were fractionated on Sephadex G100 superfine as described in the Material and Methods section. Fractions containing PLA_2_ activity are shown in red.

**Figure 6:**
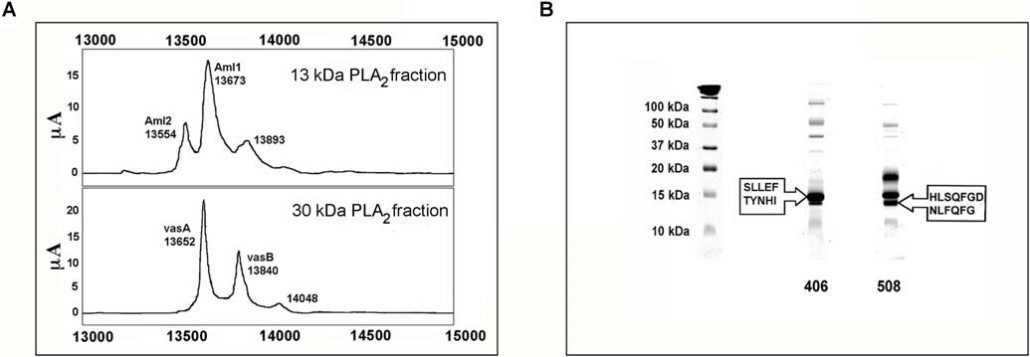
PLA_2_ analysis by SELDI and SDS-PAGE. A) *Vaa* and *Vaz* PLA_2_ fractions isolated from pooled venoms as shown in Figure 5 were spotted on WCX2 ProteinChips. Normalised mass (m/z) for each peak (in Daltons (Da)) is demonstrated on the X-axis, while intensity (μA) is plotted on the Y-axis. B) Venoms 406 and 508 were separated by SDS-PAGE on a 20% homogeneous acrylamide gel under reducing conditions. N-terminal and internal sequences were determined as described in the Material and Methods section.

SELDI-TOF-MS ProteinChip technology was used to capture and analyze proteins of 13 to 14 kDa from crude venom samples. Several ProteinChips were tested to identify the chemical interaction able to bind proteins of the expected molecular weights. Hydrophobic (H4, H50) and hydrophilic (SAX2 and WCX2) arrays were used to differentiate venom samples from *Vaa* (French department “Loire-Atlantique”) and *Vaz* (French department “Hautes-Pyrénées”) snakes (4 individual snakes for each subspecies). Best and similar profiles were obtained using cation-exchange (WCX2) and H4 (hydrophobic) chromatography surfaces. *Vaa* venoms gave 3 peaks at molecular weights between 13500 and 13900 Da, and *Vaz* venom gave 3 major peaks between 13650 and 14060 Da (Figure 4). According to the the PLA_2_ transcriptome data of venom of this two subspecies, we would expect to detect by SELDI, in this range of molecular weights, AmI1 and AmI2 in *Vaa* venoms and AmI1, vasA and vasB in *Vaz* venoms. To verify whether PLA_2_s were indeed immobilized on the SELDI chips, we first resolved pooled *Vaa* and pooled *Vaz* venoms by gel filtration on a Sephadex G75 column (Figure 5A and 5B). The main fractions presenting PLA_2_ activity (13 kDa for *Vaa* and 30 kDa for *Vaz* venom) were isolated. Proteins contained in these two fractions were separated by SDS-PAGE (not shown) and transferred onto PVDF, and stained with Amidoblack. N-terminal sequences were determined by Edman degradation. Three sequences were identified in the 13 kDa *Vaa* PLA_2_ fraction: HLSQF (AmI1, 13673 Da, gi:33187128), NLYQF (AmI2, 13553 Da, gi:33187122) and RDRPM (no hit found by Blast searches). Two sequences were identified in the 30 kDa *Vaz* PLA_2_ fraction: NLFQFGDMILQK (vasA, 13655 Da, gi:33187114) and NLFQFAKMINGK (vasB, 13841 Da, gi:33187118). The profile of these fractions was also analyzed by SELDI (Figure 6A). The *Vaa* 13 kDa PLA_2 _fraction profile gave three peaks, the molecular weight of the two first peaks (13554 Da and 13673 Da) matching those of AmI2 and AmI1 respectively; the profile of the *Vaz* 30 PLA_2_ kDa fraction (vaspin) contained two main peaks, the first (13652 Da) consistent with the molecular weight of vasA and the second (13840 Da) with the molecular weight of vasB. We also studied the PLA_2_s of individual *Vaz* venoms to test for the presence of AmI1. Venom 508 was separated by SDS-PAGE (20% acrylamide gel; Figure 6B) and transferred onto a PVDF membrane. The lowest of the two bands migrating at 14 kDa was sequenced: a mixture of 2 N-terminal sequences was obtained corresponding to AmI1 (main sequence, HLSQFGDMIN) and vasA (NLFQFG) (Figure 6B).

The PLA_2_ profiles of isolated *Vaa* and *Vaz* PLA_2_ fractions matched those of the complete venom samples; this confirms that PLA_2_s are effectively bound onto the ProteinChip surface. The first peak of *Vaz* venoms probably contains a mixture of vasA and AmI1 PLA_2_. Indeed, a pure vasA peak, as found in the *Vaz* 30 kDa PLA_2_ fraction, was thin (Figure 6A) whereas the peaks detected in *Vaz* venoms were thicker (Figure 4).

**Figure 7:**
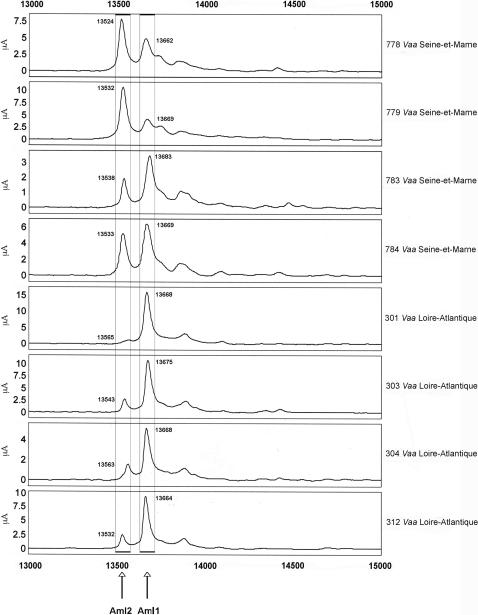
SELDI profiles of venoms from Seine-et-Marne (code no. 77) and Loire-Atlantique (code no. 44). Peaks matching the molecular weights of AmI1 and AmI2 are indicated on the spectra.

**Figure 8 pone-0001194-g008:**
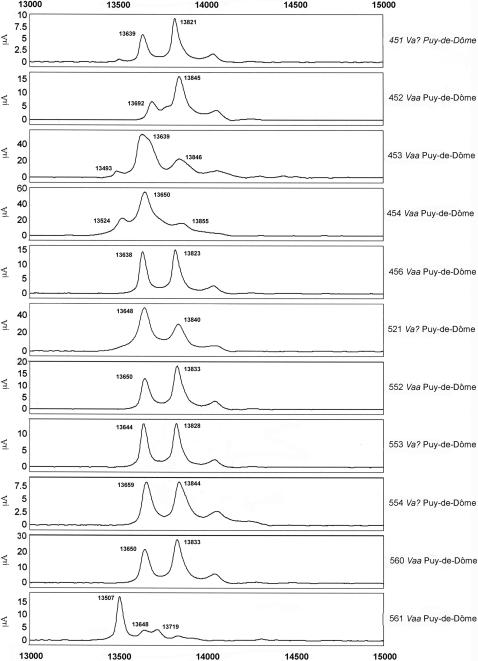
SELDI profiles of venoms from Puy-de-Dôme (code no. 63). Peak molecular weights consistent with those of PLA_2_s are indicated on the spectra.

**Figure 9 pone-0001194-g009:**
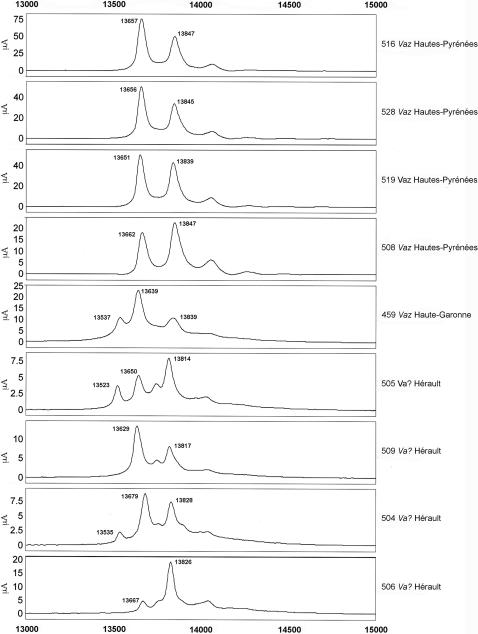
SELDI profiles of venoms from the south-west of France. Peak molecular weights consistent with those of PLA_2_s are indicated on the spectra.

**Figure 10 pone-0001194-g010:**
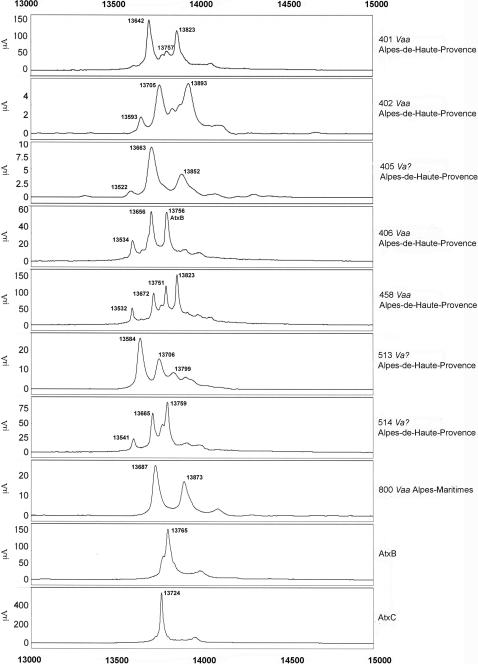
SELDI profiles of venoms from the south-east of France. Peak molecular weights consistent with those of PLA_2_s are indicated on the spectra.

**Figure 11:**
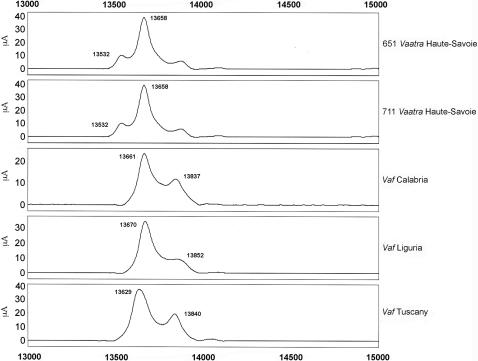
SELDI profiles of venoms from Haute-Savoie (code no. 74) and Italy. Peak molecular weights consistent with those of PLA_2_s are indicated on the spectra.

Next, we analyzed several individual venoms from snakes from French departments and neighboring countries (Figures 7–11). Venoms were grouped according to their geographical localization and/or their subspecies. *Vaa*-type venoms from the Loire-Atlantique (code no. 44) and Seine-et-Marne (code no. 77) were characterized by the presence of two main peaks consistent with the molecular weights of AmI2 and AmI1 (Figure 7). The relative abundance of these two proteins differed between the venoms of this group. Venoms collected in the Puy-de-Dôme were classified into two groups (Figure 8): the PLA_2_ profiles of 451, 456, 521, 552, 553, 554 and 560 were similar to those of *Vaz* venoms, whereas the other profiles were heterogeneous. Venoms from the south-west of France (Haute-Pyrénée, Haute-Garonne, Hérault) have diverse PLA_2_ profiles (Figure 9). *Vaz* venom 459 presents an additional peak of AmI2 not found in *Vaz* venoms from the Hautes-Pyrénées. The PLA_2_ SELDI profiles of *Va* venoms from Hérault were complex and heterogeneous: AmI2 was found in venoms 504 and 505 but not in venoms 506 and 509. Venom SELDI profiles from the south-east of France were heterogenous (Figure 10). Their complexity is in agreement with transcriptome data, which indicate the presence of AmI1, AmI2, vasA, vasB, AtxA, AtxB, and AtxC transcripts in the venom glands of some of the snakes captured in this region [30]. The SELDI profiles suggest that AmI2 is not present in venom 800. Interestingly, an additional peak of molecular weight 13751 to 13759 Da was identified in venoms 406, 458 and 514. SELDI experiments indicate that the molecular weight of AtxB is 13765 Da and AtxC has a molecular weight of 13724 Da (Figure 10). The molecular weights of AtxA and AtxB predicted from their sequences differ only by two Da, and therefore C-terminal sequencing is required to differentiate between these two isoforms. Venom 406 was separated by SDS-PAGE on a homogeneous 20% acrylamide gel (Figure 6B). The band corresponding to Atx (N-terminal sequence determined by Edman degradation after transfer onto PVDF membrane: SLLEF, Figure 6B) was cut out of the gel and digested with LysC. The peptides were separated by HPLC and the peptide corresponding to the C-terminal peptide was sequenced (TYNHI) and shown to correspond to AtxB (Figure 5D). This result is consistent with transcriptome data, which indicated that few AtxA transcripts are present in the venom glands of snake 406 [30]. SELDI profiles of Haute-Savoie *Vaatra* venoms are very similar to those of *Vaa* venoms from Loire-Atlantique (Figure 11). AmI2 appears to be absent from the venoms of *Vaf* (Figure 11).

**Figure 12:**
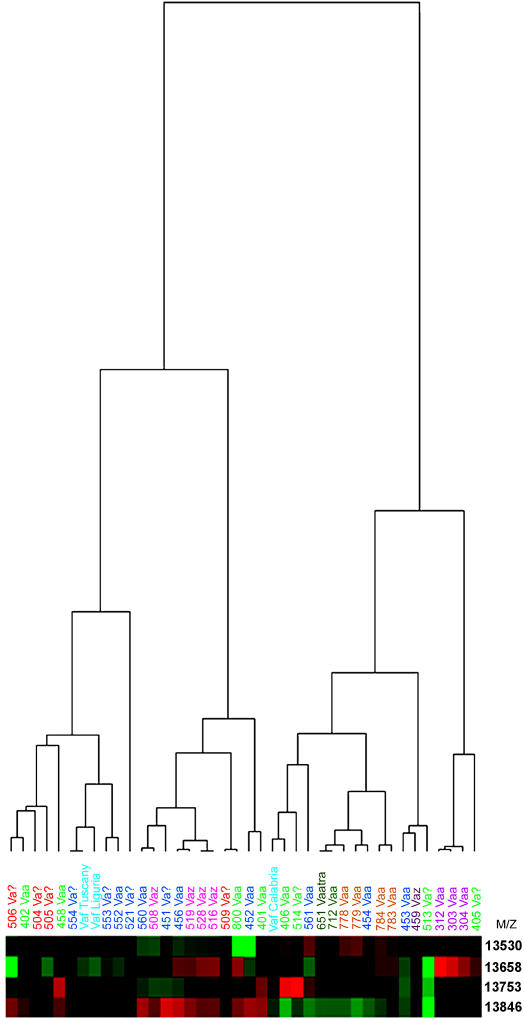
Heatmap obtained with clusters with p-values<0.05. Each column in the heatmap corresponds to a spectrum, each row to a cluster (or a potential biomarker), and each cell to a peak, with the colour indicating intensity of expression (green: low expression, red: high expression). Related spectra are sorted and grouped according to similarity, resulting in the dendrogram. The length of a branch is inversely proportional to the relatedness of the spectra. Average normalised mass (m/z) of the 4 clusters with p-values<0.05 are indicated on the right of the heatmap.

The spectra obtained were grouped into geographical clusters (cluster A: Loire-Atlantique, cluster B: Puy-de-Dôme; cluster C: Hautes-Pyrénées; cluster D: Hérault; cluster E: Haute-Garonne; cluster F: Alpes-Maritimes and Alpes-de-Haute-Provence; cluster G: Seine-et-Marne; cluster H: *Vaf* from Italy) and analyzed with CiphergenExpress software to study the relationships between the profiles. A list of 4 peaks with p-values<0.05 were selected, which helped discriminating between the various venom samples: 13530 Da (AmI2), 13658 Da (AmI1 or vasA), 13753 Da (AtxA or B) and 13846 Da (vasB). A heat map was generated to see how well groups could be separated in a non-supervised way, based on the data set provided: it gave an interesting classification of the venoms (Figure 12). According to the dendrogram obtained, the venoms are separated into two major groups. The first group is distinguished by the down-regulation of the expression of AmI2 (13530 Da) and the over-expression of vasB (13854 Da). The second group is mainly characterized by the down-regulation of the expression of vasB. All venoms of Hautes-Pyrénées and Hérault are clustered into the first group, whereas all venoms from Loire-Atlantique, Seine-et-Marne and Haute-Savoie are found in the second group. Eight out of 11 venoms collected in Puy-de-Dôme are clustered in the first group whereas venoms from Alpes-Maritimes and Alpes-de-Haute-Provence are distributed in a homogeneous way in the two groups. A heterogeneity is also noted for *Vaf* venoms, which are distributed into the two groups (Figure 12). Venoms 401, 406, 458 and 514 were clearly characterized by the over-expression of a peak at an average molecular weight of 13753 Da. This is consistent with the molecular weight of AtxB since we have shown that the level of expression of AtxA is very low in the venom glands of snakes 406 and 514 [30] and only AtxB was detected in venom 406 by microsequencing (Figure 6B).

### Immunome of snake venoms

Cross-reactions to Atx antibodies were previously shown to identify the presence of neurotoxins (Atx and/or vaspin) in the blood of patients presenting neurological symptoms following snake bite by vipers in “Alpes-Maritimes” and “Alpes-de-Haute-Provence” (south-east of France) [18]. The same approach was therefore used to identify snakes that express neurotoxins in their venom (Table 3). *Vaa* venoms from Loire-Atlantique, Seine-et-Marne and Haute-Savoie did not show any cross-reaction with anti-Atx antibodies consistent with the absence of neurological cases of envenomation in these areas (Table 3). *Vu* venom 620 (Table 3) and *Vu* venoms from Alpes-Maritimes (data not shown) did not cross-react with Atx, showing that vaspin expression is low or inexistent. Venoms from Puy-de-Dôme had diverse cross-reactivities ranging from 0 to 2 depending on their area of capture. A higher heterogeneity was observed among venoms from Alpes-de-Haute-Provence with cross-reactivities ranging from 0 to 4. The Atx cross-reaction scores for snake venoms from Hautes-Pyrénées were between 1 and 3. Venoms from Hérault and Haute-Garonne were all highly reactive with anti-Atx antibodies. We also tested the cross-reactivities of *Vaz* venom samples collected in other departments of the south-west of France: Tarn (code no. 81), Aveyron (code no. 12), Pyrénées-Atlantique (code no. 64), Aude (code no. 11) and Pyrénées Orientales (code no. 66). They all presented maximum scores of cross-reactions (data not shown). Venoms from *Vll* from Burgos (Spain) did not show any cross-reactivity with anti-Atx antibodies, consistent with the PLA_2_ composition of the *Vll* genome, which includes only AmI1 and AmI2 genes. *Vaf* venoms collected in Tuscany, Liguria and Calabria (Italy) showed Atx cross-reactivity scores of 1 to 3.

**Table 3:** Evaluation of neurotoxicity of venoms collected in France by measuring cross-reactivity to AtxA antibodies.

Label number	Administrative department	Viper identification	Cross-reaction with AtxA
402, 403, 404	Alpes-de-Haute-Provence (04)	*Vaa*	0
405	Alpes-de-Haute-Provence (04)	*Va ?*	0
651	Haute-Savoie (74)	*Vaatra*	0
712, 713,714, 715, 716	Haute-Savoie (74)	*Vaa*	0
564, 602	Hérault (34)	*Vaa «garrigue»*	0
301, 303, 304, 312	Loire-Atlantique (44)	*Vaa*	0
452, 453, 454, 455, 501, 502, 503, 551, 601, 561	Puy-de-Dôme (63)	*Vaa*	0
778, 779, 783, 784	Seine-et-Marne (77)	*Vaa*	0
620	Vaucluse (84)	*Vu*	0
800	Alpes-Maritimes (06)	*Vaa*	1
508, 519	Hautes-Pyrénées (65)	*Vaz*	1
552, 559	Puy-de-Dôme (63)	*Vaa*	1
510, 512, 521, 553, 554	Puy-de-Dôme (63)	*Va ?*	1
513	Alpes-de-Haute-Provence (04)	*Va ?*	2
613	Gironde (33)	*Vaz*	2
516, 528	Hautes-Pyrénées (65)	*Vaz*	2
451	Puy-de-Dôme (63)	*Va?*	2
456, 560	Puy-de-Dôme (63)	*Vaa*	2
527	Hautes-Pyrénées (65)	*Vaz*	3
401, 406, 458	Alpes-de-Haute-Provence (04)	*Vaa*	4
514	Alpes-de-Haute-Provence (04)	*Va?*	4
603	Alpes-Maritimes (06)	*Vaa*	4
565	Gironde (33)	*Vaz*	4
459	Haute-Garonne (31)	*Vaz*	4
504, 505, 506, 509	Hérault (34)	*Va?*	4

Cross-reactions to AtxA antibodies were tested by ELISA as described in the Material and Methods section. Optical densities measured at dilutions of anti-AtxA IgG of 1/16,000 were used for cross-reactivity gradation: level 0: OD≤0.1, level 1: 0.1<OD≤0.2; level 2: 0.2<OD≤0.3; level 3: 0.3<OD≤0.4; level 4: OD>0.4.

Interestingly, all venoms from SELDI group 1 but one (452) had Atx cross-reactivities ranging from 1 to 4 whereas 73% of venom from group 2 did not cross-react with Atx. This observation shows that the SELDI technology is definitely efficient to differentiate *Vaz*-type neurotoxic venoms from classical ones. Neurotoxic atypical venoms like those from the south-east of France are less easily identified unless they clearly over-express Atx, which can be easily identified in the heat map.

### Geographical distribution of neurotoxic snakes venoms and cases of envenomation

**Figure 13:**
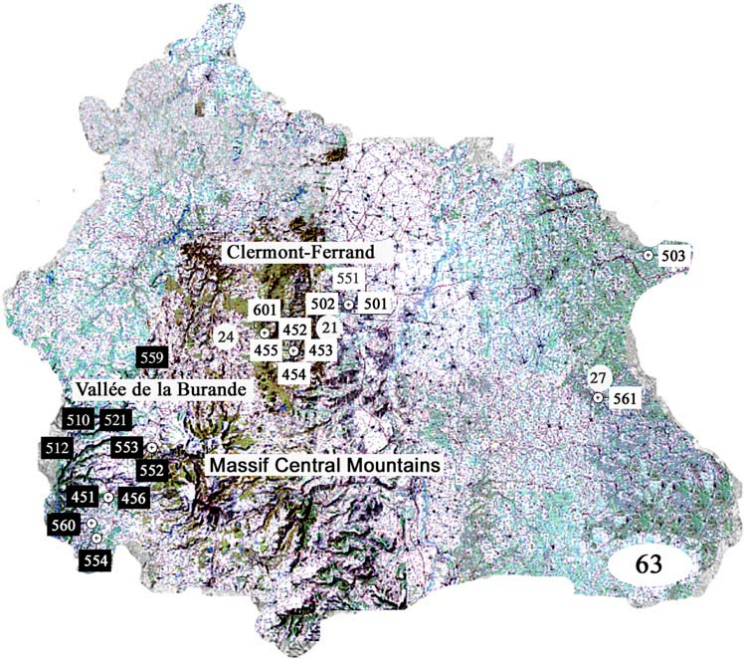
Snake venom characteristics and type of grade 2 and 3 envenomations observed in Puy-de-Dôme (code no. 63). Envenomation cases are presented in circles and snake venoms in rectangles. Classical venoms or envenomations cases are in white and neurotoxic venoms and envenomations are reported in black.

**Figure 14:**
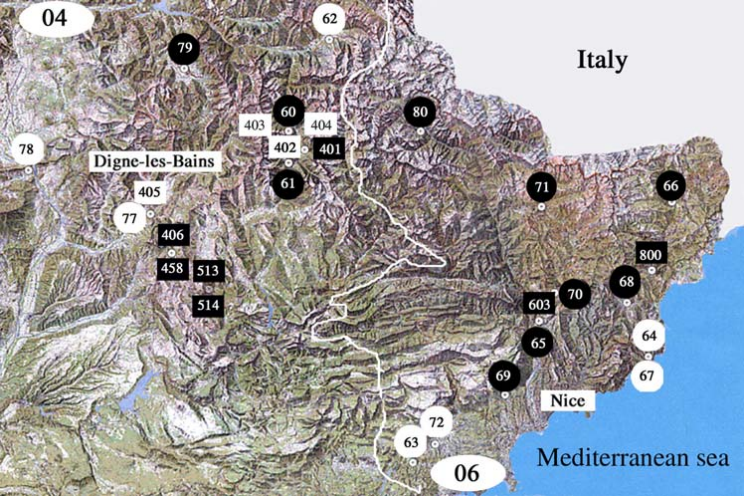
Snake venom characteristics and type of grade 2 and 3 envenomations observed in Alpes-de-Haute-Provence (code no. 04) and Alpes-Maritimes (code no. 06). Envenomation cases are presented in circles and snake venoms in rectangles. Classical venoms or envenomations cases are in white and neurotoxic venoms and envenomations are reported in black. The white line indicates the border between the two deparments.

The unusual features of immunological cross-reactions observed in Puy-de-Dôme, Alpes-Maritimes and Alpes-de-Haute-Provence as well as the complexity of their classification according to their proteomic profile prompted us to compare the geographical distribution of snake venom data and cases of envenomation. The *Vipera aspis* vipers of Puy-de-Dôme whose venom belong to SELDI group 1 (CiphergenExpress) and cross-react with Atx are grouped in the south-west of the department (Figure 13). These venoms were classified in the same group as that of *Vaz* venoms from Hautes-Pyrénées. Interestingly, only classical features of *Vipera aspis* envenomations were observed for patients collected at Clermont-Ferrand hospital. However, it must be specified that the localization of the geographical sector where the bite took place is not specified by the epidemiologic investigation. The distribution was more complex in Alpes-Maritimes and Alpes-de-Haute-Provence (Figure 14). In Alpes-Maritimes, all snakes and all envenomation cases were neurotoxic as far as they were located at distance of the mediterranean coast. In Alpes-de-Haute-Provence, an equal number of neurotoxic and non-neurotoxic venoms and envenomations were reported. At Digne-les-Bains, a classical envenomation was observed and a non-neurotoxic viper was captured. At Colmars-les-Alpes, a neurotoxic case was reported whereas both neurotoxic and non-neurotoxic snakes were captured.

## Discussion

Cases of neurological envenomation by *Vipera aspis* snakes have regularly been reported since 1992 in the south-east of France (Alpes-Maritimes and Alpes-de-Haute-Provence). The presence of genes encoding PLA_2_ neurotoxins in the *Vaa* snake genome led us to conduct an epidemiological survey of snake bite in various regions of France. Neurological signs were reported after snake bite in three regions of France: Languedoc-Roussillon, Midi-Pyrénées and PACA. All cases involved ptosis, which appears as a characteristic sign of neurotoxic *Vipera aspis* envenomations. The time to between envenomation and the onset of symptoms was variable, but symptom onset was often late. Ptosis and ophtalmoplegia, two of most frequent symptoms, were shown to occur after 9.03±5.6 hrs and 10±6.12 hrs respectively.

The clinical symptoms observed in “Alpes-Maritimes” and “Aveyron” were the most diverse, with nine different neurological signs. The diversity of symptoms observed was not related to the severity of envenomation, or to the age of the patients. Moreover, similar symptoms were observed after envenomation by venoms containing vaspin or ammodytoxin. This was true whether these toxins acted at the presynaptic part of the neuromuscular junction, like the momomeric ammodytoxin, or bound to the postsynaptic part, like heterodimeric vaspin.

We used several approaches to characterize the venom PLA_2_ composition of snakes captured in the areas in which the epidemiological survey was performed. We analyzed the genes and transcripts encoding venom PLA_2_s. We used SELDI technology to study the diversity of PLA_2_ neurotoxins in various venom samples. Electrospray and MALDI-MS is a faster, more accurate approach than SDS-PAGE for the characterization of venom components [31], [32]. SELDI-TOF-MS can be considered as an extension of the matrix-assisted laser desorption/ionization (MALDI)-TOF-MS method [33]. In the SELDI method, protein solutions are applied to the spots of ProteinChip Arrays, which have been derivatized with planar chromatographic chemistries. The proteins actively interact with the chromatographic array surface, and become sequestered as well as separated from salts and other sample contaminants by subsequent on-spot washing with appropriate buffer solutions. Prefractionation, a sample preparation prerequisite that complicates the MALDI analysis, often resulting in sample loss as well as artifactual qualitative and quantitative variances, is therefore not required for SELDI analyses. This is particularly interesting if one works with samples of which the quantities are reduced. In parallel, we evaluated venom neurotoxicity by analyzing cross-reactivity with anti-Atx antibodies. We have previously used this method to detect neurotoxins in the blood of patients bitten by vipers in the south-east of France who presented neurological symptoms [18]. Three snake populations were identified as worthy of particular attention based on their neurotoxic venom PLA_2_ content.

The genomic analysis of snakes captured in the south-east of France revealed considerable diversity in the genes encoding PLA_2_. Genes encoding two types of neurotoxin, Atx isoforms and vaspin, were identified. These genes were not constantly expressed, as shown by transcriptome data. Consistent with this finding, venom SELDI profiles and neurotoxin production were highly variable and diverse. This finding is consistent with the substantial variability of symptoms in this region, in which both neurotoxic and classical envenomations are reported (Figure 14).

The genomes of *Vaz* snakes from Gironde and Hautes-Pyrénées were characterized by the absence of the AmI2 gene. Venom PLA_2_ profiles were highly homogeneous. All contained neurotoxic PLA_2_s, regardless of where the snakes were captured. However, neurotoxin levels varied between venom samples, with the lowest level of variability for snakes from the center of the range of distribution and the highest level of variability for snakes collected in zones of intergradation between *Vaz* and *Vaa* snakes.

The genomes of *Vaa* snakes from Puy-de-Dôme were very similar to those of *Vaa* snakes from Pays-de-la-Loire, but one snake did not possess an AmI2 gene; interestingly, vaspin transcripts were detected in this snake only. SELDI profiles of venoms from Puy-de-Dôme revealed that PLA_2_ composition was related to the geographic location of the corresponding snake. Venoms from the west of the department were identical to *Vaz* venoms, and contained vaspin. Those from the center and east of the department displayed heterogeneous profiles, and no cross-reaction with anti-Atx antibodies was reported.

Our results raise two issues concerning the clinical expression of venom neurotoxicity and the origin of the two neurotoxic populations of *Vaa*.

**Figure 15:**
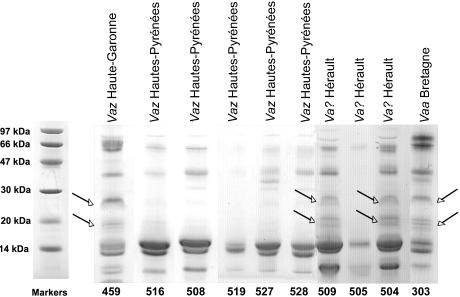
Hybrid SDS-PAGE venom profiles from snakes captured in Hérault (code no. 34) and Haute-Garonne (code no. 31). Venoms 504, 505, and 509 from Hérault, 459 from Haute-Garonne, *Vaz*-type venoms 508, 516, 519, 527, 528 from Hautes-Pyrénées and *Vaa*-type venom 303 from Loire-Atlantique were separated by a 20% SDS-PAGE under denaturating and reducing condtitions. Molecular weight markers are shown on the left. Arrows indicate protein bands specifically found in *Vaa* venoms.

A correlation was found between the expression of neurological symptoms in humans and the intensity of the cross-reaction of venoms with anti-Atx antibodies, which is correlated with the level of neurotoxin expression. Neurotoxic envenomations were reported only in the areas with the highest scores of Atx cross-reactivity. Atx cross-reactivity scores of 4 are frequent for snake venoms in the south-east of France, where neurotoxic envenomations have been regularly reported (Supplementary Table S2, Figure 14). No such score was ever reported for snakes from Puy-de-Dôme and only “classical” symptoms of *Vaa* snake envenomations were observed in this area (Supplementary Table S2, Figure 13). This was also true in the neighboring departments to the west: Creuse (code no. 23), and Corrèze (code no. 19) (hospital of Ussel, personnal communication). Atx cross-reactivity scores of 4 were mainly observed on the periphery of the distribution range of *Vaz* snakes. No neurological symptoms associated with *Vaz* snake bites were described in a retrospective survey of 240 *Vaz* envenomations during the last 40 years in the south-west of France [34]. Another retrospective survey performed between 1995 and 2000 confirmed the absence of neurological signs following *Vaz* bites in the Aquitaine region [35]. The neurological symptoms reported after one *Vaz* bite in this region were mild and not convincing according to our criteria [36]. The patient suffered only drowsiness and muscle weakness, with no ptosis. Thus, neurological signs including ptosis have only been observed in Hérault and Aveyron (as previously described by [37]). Venoms tested in these departments also presented the highest scores for Atx cross-reactivity. Thus, the amount of vaspin was shown to vary between snakes. Intergradation areas were sites of high levels of vaspin expression. Snakes captured in Haute-Garonne and Languedoc-Roussillon are probably hybrids between *Vaa* and *Vaz* snakes, as suggested by the following factors: 1) difficulties in identifying clearly their subspecies; 2) the heterogeneity of their PLA_2_ SELDI profiles, which demonstrated the expression of AmI2 in the venom of two snakes; 3) the composition of their venoms that present by SDS-PAGE bands specific of *Vaa* venoms as shown in Figure 15.

We will now consider the origin of the two neurotoxic populations of *Vaa*. Two data sets must be considered for the southeastern neurotoxic snake population: 1) the regular reports of neurotoxic envenomations in south-east France since 1992 suggesting the recent appearance of a new snake population and 2) the presence of genes encoding Atx isoform genes and Bov-B line retroposons in the genome of this neurotoxic snake population. We found published reports of envenomations with neurological signs in four departments of PACA: 1) Alpes-Maritimes [38], 2) Alpes-de-Haute-Provence [39], [40], 3) Hautes-Alpes, and 4) Vaucluse [41]. The case from Alpes-de-Haute-Provence [40] was wrongly attributed to *Vipera ursinii*, which can clearly be excluded in light of recent observations [42]. These data indicate that the neurotoxic *Vipera aspis* population of the south-east of France did not appear in the nineties. Thus, regular report of neurotoxic snake bites in south-east France may be explained either by the careful survey performed by the Poison Center of Marseilles since the start of the 1990's, which could have highlighted neurotoxic snake bites or to the modification of the neurotoxic snake population behaviour. Interestingly, the epidemiological survey has indicated that most of the neurotoxic snake bites occurred near dwellings or inside towns. The consecutive rarefaction of the preys to forest fires and to the drought that has prevailed for a few years in the south-east of France could explain this phenomenon.

We analyzed the occurrence of neurotoxic envenomations in neighboring countries. We also analyzed the genome, transcriptome and proteome characteristics of snake venoms in these countries. Neurological cases attributed to *Vaf* snake bites were reported in Italy from 1989 to 1995 [43], [44]. The symptoms described, and their timing did not significantly differ from our observations. The genome of the *Vaf* captured in Piemont (Italy) contains vaspin genes, but no Atx genes. *Vaatra* is present in Italy, at the border with France. This species is also present in Switzerland, but no case of neurotoxic envenomation associated with *Vaatra* snake bite has ever been reported by the Swiss National Poison Center of Zurich (personal communication). These observations are consistent with our genomic analysis indicating that the *Vaatra* genome contains no genes encoding neurotoxins. We suggest that the French neurotoxic snake population has resulted from hybridization between *Vipera aspis* and *Vipera ammodytes*. The following observations support this view: 1) natural hybrids between *Vaf* and *Vam rufoi* can be found in the north-east of Italy, where these two species cohabit, 2) a phylogenetic analysis of *V. aspis* snakes has shown that “neurotoxic” *Vaa* collected in south-eastern France belong to a phylogenetic group separate from that containing the other French specimens of this subspecies [27]. According to epidemiological data, the neurotoxic snake population spans the territory of at least four departments. Given the low dispersion rate of these snakes, the hybridization event was probably not recent and may have occurred in refuge areas during the last period of glaciation. The Southern Alps (Italy) served as refuge areas for *V. aspis* and *V. ammodytes* snakes during several ice ages [45], [46]. Moreover, analysis of the phylogeography of *V. aspis* has suggested the existence of refuge areas in south-eastern France [47]. This is consistent with the presence of Atx genes and Bov-b line retroposons in the genomes of *Vaf* captured in the south of Italy, and of *Vammon* from Bulgaria.

The origin of the neurotoxic population of Puy-de-Dôme appears different. All neurotoxic individuals are clustered in the south-west part of the department, near the tributaries of the Dordogne River, which flow into the Gironde. These snakes may have originated from the *Vaz* population of Gironde. They may have colonized northern areas from the ice refuges of the south-west of France, by moving along the rivers, which constitute thermal corridors. Their PLA_2_ profile is consistent with this possibility, because their genomes, like those of *Vaz* snakes, contain no AmI2 gene. Our analysis of PLA_2_ SELDI profiles suggests that the area of distribution of this potentially neurotoxic snake population to the east may be limited towards the west by the Massif Central Mountains. More studies must be performed on snake venoms from the east part of the department to define the range of this neurotoxic population.

Fry *et al*. recently reconsidered the very concept of “non-venomous” snakes after studying the transcriptomes of the venom gland of non-front-fanged snakes: these transcriptomes contain sequences encoding several toxin types [48], [49]. Similarly, our study shows that we have to reconsider *Vipera aspis aspis* as a “cryptoneurotoxic” species. Its genome contains an arsenal of PLA_2_ neurotoxins ready to be expressed under stimuli that still need to be identified. Our work demonstrates that a multidisciplinary approach can fully characterize the diversity in snake venom composition and identify its medical and public health consequences. Thus, by combining epidemiological data concerning snake bites with genomic, tanscriptomic, proteomic and immunochemical analyses of the major toxic components of venoms, we were able to provide a clear description of the potential neurotoxicity of *Vipera aspis* bites in France. These observations as a whole illustrate the biodiversity of venoms of the genus *Vipera*. The geographical variations in the composition of *Vipera aspis* venom have major consequences for the preparation of neutralizing antivenoms [50], [51]. Indeed, it strengthens the arguments in support of the use of polyvalent antivenoms able to neutralize the venom of *Vipera aspis*, *Vipera berus* and *Vipera ammodytes* in the treatment of viper envenomations in France.

## Supporting Information

Table S1 (0.17 MB DOC)

Table S2 (0.24 MB DOC)
