# Supplementary material for: Reappraisal of Vipera aspis Venom Neurotoxicity
Source: PLoS One. 2007 Nov 21;2(11):e1194. doi: 10.1371/journal.pone.0001194 (PMC2065900; doi:10.1371/journal.pone.0001194)
Supplement: Table S1 — (0.17 MB DOC) [file pone.0001194.s001.doc]

Table 1: Description of the snakes captured in France

| **Label Number** | **Region** | **Department** | Viper identification | | | | |
| --- | --- | --- | --- | --- | --- | --- | --- |
|  |  |  | **Viper** | **Age** | **Sex** | **Altitude (m)** | **Place of capture** |
| 565 | Aquitaine | Gironde (33) | *Vaz* | Adult | Male | 33 | Etang de Cazaux |
| 613 | Aquitaine | Gironde (33) | *Vaz* | Sub-adult | Male | 33 | Etang de Cazaux |
| 451 | Auvergne | Puy-de-Dôme (63) | *Va?* | Adult | Male | 740 | Vallée de la Burande |
| 452 | Auvergne | Puy-de-Dôme (63) | *Vaa* | Adult | Male | 970 | Puy de Manson |
| 453 | Auvergne | Puy-de-Dôme (63) | *Vaa* | Adult | Female | 970 | Puy de Manson |
| 454 | Auvergne | Puy-de-Dôme (63) | *Vaa* | Adult | Male | 975 | Puy de Manson |
| 455 | Auvergne | Puy-de-Dôme (63) | *Vaa* | Adult | Female | 970 | Puy de Manson |
| 456 | Auvergne | Puy-de-Dôme (63) | *Vaa* | Juvenile | Female | 740 | Vallée de la Burande |
| 501 | Auvergne | Puy-de-Dôme (63) | *Vaa* | Adult | Male | 370 | Clermont-Ferrand |
| 502 | Auvergne | Puy-de-Dôme (63) | *Vaa* | Adult | Female | 370 | Clermont-Ferrand |
| 503 | Auvergne | Puy-de-Dôme (63) | *Vaa* | Adult | Female | 870 | Ferrières |
| 510 | Auvergne | Puy-de-Dôme (63) | *Va?* | Adult | Female | 750 | Chavanon |
| 512 | Auvergne | Puy-de-Dôme (63) | *Va?* | Sub-adult | Female | 750 | Chavanon |
| 521 | Auvergne | Puy-de-Dôme (63) | *Va?* | Adult | Female | 750 | Chavanon |
| 551 | Auvergne | Puy-de-Dôme (63) | *Vaa* | Adult | Male | 370 | Clermont-Ferrand |
| 552 | Auvergne | Puy-de-Dôme (63) | *Vaa* | Adult | Male | 920 | St-Sauves-d'Auvergne |
| 553 | Auvergne | Puy-de-Dôme (63) | *Va?* | Adult | Female | 920 | St-Sauves-d'Auvergne |
| 554 | Auvergne | Puy-de-Dôme (63) | *Va?* | Adult | Male | 725 | Cros |
| 559 | Auvergne | Puy-de-Dôme (63) | *Vaa* | Adult | Female | 530 | Tingaud |
| 560 | Auvergne | Puy-de-Dôme (63) | *Vaa* | Adult | Female | 840 | Maurissoux |
| 561 | Auvergne | Puy-de-Dôme (63) | *Vaa* | Juvenile | Female | 425 | Olliergues |
| 601 | Auvergne | Puy-de-Dôme (63) | *Vaa* | Adult | Female | 975 | Puy de Manson |
| 778 | Ile-de-France | Seine-et-Marne (77) | *Vaa* | Adult | Male | 70 | Forêt de Fontainebleau |
| 779 | Ile-de-France | Seine-et-Marne (77) | *Vaa* | Adult | Male | 70 | Forêt de Fontainebleau |
| 783 | Ile-de-France | Seine-et-Marne (77) | *Vaa* | Juvenile | nd | 70 | Forêt de Fontainebleau |
| 784 | Ile-de-France | Seine-et-Marne (77) | *Vaa* | Juvenile | nd | 70 | Forêt de Fontainebleau |
| 504 | Languedoc-Roussillon | Hérault (34) | *Va?* | Sub-adult | Female | 627 | St-Pierre-la-Fage |
| 506 | Languedoc-Roussillon | Hérault (34) | *Va?* | Sub-adult | Male | 627 | St-Pierre-la-Fage |
| 505 | Languedoc-Roussillon | Hérault (34) | *Va?* | Adult | Female | 627 | St-Pierre-la-Fage |
| 509 | Languedoc-Roussillon | Hérault (34) | *Va?* | Adult | Female | 627 | St-Pierre-la-Fage |
| 564 | Languedoc-Roussillon | Hérault (34) | *Vaa* | Adult | Male | 600 | Pic St- Loup |
| 602 | Languedoc-Roussillon | Hérault (34) | *Vaa* | Juvenile | Female | 600 | Pic St- Loup |
| 459 | Midi-Pyrénées | Haute-Garonne (31) | *Vaz* | Juvenile | Male | 235 | Toulouse |
| 508 | Midi-Pyrénées | Hautes-Pyrénées (65) | *Vaz* | Adult | Male | 420 | Lourdes |
| 516 | Midi-Pyrénées | Hautes-Pyrénées (65) | *Vaz* | Sub-adult | Male | 420 | Lourdes |
| 519 | Midi-Pyrénées | Hautes-Pyrénées (65) | *Vaz* | Adult | Female | 475 | Capvern |
| 527 | Midi-Pyrénées | Hautes-Pyrénées (65) | *Vaz* | Juvenile | Female | 310 | Pic du midi |
| 528 | Midi-Pyrénées | Hautes-Pyrénées (65) | *Vaz* | Sub-adult | Female | 475 | Capvern |
| 401 | PACA | Alpes-de-Haute-Provence (04) | *Vaa* | Adult | Male | 1455 | Colmars-les-Alpes |
| 402 | PACA | Alpes-de-Haute-Provence (04) | *Vaa* | Juvenile | nd | 1465 | Colmars-les-Alpes |
| 403 | PACA | Alpes-de-Haute-Provence (04) | *Vaa* | Juvenile | nd | 1465 | Colmars-les-Alpes |
| 404 | PACA | Alpes-de-Haute-Provence (04) | *Vaa* | Juvenile | nd | 1465 | Colmars-les-Alpes |
| 405 | PACA | Alpes-de-Haute-Provence (04) | *Va?* | Sub-adult | Female | 730 | Digne-les-Bains |
| 406 | PACA | Alpes-de-Haute-Provence (04) | *Vaa* | Adult | Female | 1154 | Chaudon-Norante |
| 458 | PACA | Alpes-de-Haute-Provence (04) | *Vaa* | Adult | Male | 914 | Chaudon-Norante |
| 513 | PACA | Alpes-de-Haute-Provence (04) | *Va?* | Juvenile | Male | 1500 | Barreme |
| 514 | PACA | Alpes-de-Haute-Provence (04) | *Va?* | Sub-adult | Female | 1500 | Barreme |
| 603 | PACA | Alpes-Maritimes (06) | *Vaa* | nd | nd | 569 | Levens |
| 800 | PACA | Alpes-Maritimes (06) | *Vaa* | Adult | Female | 312 | Breuil-la-Roya |
| 620 | PACA | Vaucluse (84) | *Vu* | nd | nd | 1350 | Mont Ventoux |
| 301 | Pays-de-la-Loire | Loire-Atlantique (44) | *Vaa* | Adult | Male | 15 | Ste-Lucie-sur-Loire |
| 303 | Pays-de-la-Loire | Loire-Atlantique (44) | *Vaa* | Adult | Male | 15 | Ste-Lucie-sur-Loire |
| 304 | Pays-de-la-Loire | Loire-Atlantique (44) | *Vaa* | Juvenile | nd | 15 | Ste-Lucie-sur-Loire |
| 311 | Pays-de-la-Loire | Loire-Atlantique (44) | *Vaa* | Juvenile | nd | 3 | St-Aignan-Grand Lieu |
| 312 | Pays-de-la-Loire | Loire-Atlantique (44) | *Vaa* | Adult | nd | 12 | St-Géréon |
| 313 | Pays-de-la-Loire | Loire-Atlantique (44) | *Vaa* | Juvenile | nd | 12 | St-Géréon |
| 314 | Pays-de-la-Loire | Loire-Atlantique (44) | *Vaa* | Juvenile | nd | 12 | St-Géréon |
| 317 | Pays-de-la-Loire | Loire-Atlantique (44) | *Vaa* | Adult | Male | 12 | La-Chapelle-sur-Erdre |
| 318 | Pays-de-la-Loire | Loire-Atlantique (44) | *Vaa* | Adult | Female | 35 | La-Chapelle-sur-Erdre |
| 320 | Pays-de-la-Loire | Loire-Atlantique (44) | *Vaa* | nd | nd | 25 | Couëron |
| 651 | Rhône-Alpes | Haute-Savoie (74) | *Vaatra* | Adult | Male | 1000 | Bernex |
| 711 | Rhône-Alpes | Haute-Savoie (74) | *Vaatra* | Adult | Male | 1000 | Bernex |
| 712 | Rhône-Alpes | Haute-Savoie (74) | *Vaa* | Adult | Female | 1100 | Chapelle d’Abondance |
| 713 | Rhône-Alpes | Haute-Savoie (74) | *Vaa* | Adult | Female | 560 | Saint-Cergues |
| 714 | Rhône-Alpes | Haute-Savoie (74) | *Vaa* | Adult | Female | 560 | Saint-Cergues |
| 715 | Rhône-Alpes | Haute-Savoie (74) | *Vaa* | Adult | Male | 1100 | Chapelle d’Abondance |
| 716 | Rhône-Alpes | Haute-Savoie (74) | *Vaa* | Juvenile | nd | 1100 | Chapelle d’Abondance |

For each snake captured, a questionnaire was filled in by herpetologists. Snakes were identified by use of classical keys. When identification

could not be performed at the level of subspecies, a question mark was used. PACA stands for Provence-Alpes-Côte-d’Azur.
