# Supplementary material for: Reappraisal of Vipera aspis Venom Neurotoxicity
Source: PLoS One. 2007 Nov 21;2(11):e1194. doi: 10.1371/journal.pone.0001194 (PMC2065900; doi:10.1371/journal.pone.0001194)
Supplement: Table S2 — (0.24 MB DOC) [file pone.0001194.s002.doc]

Table 2: Epidemiological and clinical analyses of snakebites

| **Region** | **Administrative department** | **Case number** | **age** | **sex** | **date** | **Location** | **Grade** | **Neurological signs** |
| --- | --- | --- | --- | --- | --- | --- | --- | --- |
| Aquitaine | Gironde (33) | 1 | 11 | M | Sep-02 | Bordeaux | 2 | no |
| Auvergne | Haute-Loire (43) | 2 | 69 | F | Jun-96 | Puy-en-Velay | 3 | no |
| Auvergne | Puy-de-Dôme (63) | 3 | 50 | nm | Jul-97 | nm | 1 | no |
| Auvergne | Puy-de-Dôme (63) | 4 | 33 | nm | May-98 | nm | 1 | no |
| Auvergne | Puy-de-Dôme (63) | 5 | 35 | M | Jul-99 | nm | 1 | no |
| Auvergne | Puy-de-Dôme (63) | 6 | 23 | M | May-99 | nm | 1 | no |
| Auvergne | Puy-de-Dôme (63) | 7 | 10 | F | Jul-97 | nm | 2 | no |
| Auvergne | Puy-de-Dôme (63) | 8 | 14 | F | Sep-94 | nm | 2 | no |
| Auvergne | Puy-de-Dôme (63) | 9 | 15 | nm | May-93 | nm | 2 | no |
| Auvergne | Puy-de-Dôme (63) | 10 | 24 | M | Aug-98 | nm | 2 | no |
| Auvergne | Puy-de-Dôme (63) | 11 | 27 | nm | Apr-97 | nm | 2 | no |
| Auvergne | Puy-de-Dôme (63) | 12 | 29 | M | Jul-96 | nm | 2 | no |
| Auvergne | Puy-de-Dôme (63) | 13 | 29 | nm | Aug-95 | nm | 2 | no |
| Auvergne | Puy-de-Dôme (63) | 14 | 35 | nm | Aug-99 | nm | 2 | no |
| Auvergne | Puy-de-Dôme (63) | 15 | 45 | M | Jul-00 | nm | 2 | no |
| Auvergne | Puy-de-Dôme (63) | 16 | 45 | F | Oct-93 | nm | 2 | no |
| Auvergne | Puy-de-Dôme (63) | 17 | 45 | nm | Apr-91 | nm | 2 | no |
| Auvergne | Puy-de-Dôme (63) | 18 | 49 | M | Jul-95 | nm | 2 | no |
| Auvergne | Puy-de-Dôme (63) | 19 | 50 | M | May-99 | nm | 2 | no |
| Auvergne | Puy-de-Dôme (63) | 20 | 56 | nm | Aug-94 | nm | 2 | no |
| Auvergne | Puy-de-Dôme (63) | 21 | 71 | nm | Sep-99 | Chamalières | 2 | no |
| Auvergne | Puy-de-Dôme (63) | 22 | 73 | nm | May-99 | nm | 2 | no |
| Auvergne | Puy-de-Dôme (63) | 23 | nm | nm | Aug-94 | nm | 2 | no |
| Auvergne | Puy-de-Dôme (63) | 24 | 11 | M | Jul-95 | Aydat | 3 | no |
| Auvergne | Puy-de-Dôme (63) | 25 | 42 | M | Aug-95 | nm | 3 | no |
| Auvergne | Puy-de-Dôme (63) | 26 | 42 | M | nm | nm | 3 | no |
| Auvergne | Puy-de-Dôme (63) | 27 | 58 | F | Jul-93 | Olliergues | 3 | no |
| Auvergne | Puy-de-Dôme (63) | 28 | 64 | M | May-91 | nm | 3 | no |
| Auvergne | Puy-de-Dôme (63) | 29 | 66 | F | Sep-97 | nm | 3 | no |
| Auvergne | Puy-de-Dôme (63) | 30 | 66 | F | Jul-96 | nm | 3 | no |
| Auvergne | Puy-de-Dôme (63) | 31 | 73 | M | Sep-93 | nm | 3 | no |
| Auvergne | Puy-de-Dôme (63) | 32 | 91 | M | Apr-96 | nm | 3 | no |
| Languedoc-Roussillon | Aude (11) | 33 | 42 | F | Aug-00 | Narbonne | 1 | no |
| Languedoc-Roussillon | Gard (30) | 34 | 30 | M | Jul-01 | Quissac | 1 | no |
| Languedoc-Roussillon | Pyrénées-Orientales (66) | 35 | 53 | F | Aug-00 | Perpignan | 1 | no |
| Languedoc-Roussillon | Aude (11) | 36 | 6 | M | Jul-98 | Narbonne | 2 | no |
| Languedoc-Roussillon | Gard (30) | 37 | 3 | M | Jul-98 | Bagnols-sur-Ceze | 2 | no |
| Languedoc-Roussillon | Gard (30) | 38 | 12 | M | Jun-98 | Ales | 2 | no |
| Languedoc-Roussillon | Gard (30) | 39 | 31 | M | May-99 | Cavillargues | 2 | no |
| Languedoc-Roussillon | Hérault (34) | 40 | 5 | nm | Jun-00 | Ganges | 2 | yes |
| Languedoc-Roussillon | Hérault (34) | 41 | 40 | F | May-02 | Ganges | 2 | no |
| Languedoc-Roussillon | Hérault (34) | 42 | 23 | H | Aug-99 | Beziers | 2 | no |
| Languedoc-Roussillon | Pyrénées-Orientales (66) | 43 | 46 | M | May-01 | Osseja | 2 | no |
| Languedoc-Roussillon | Lozère (48) | 44 | 31 | M | Jun-98 | Mende | 2 | no |
| Languedoc-Roussillon | Aude (11) | 45 | 12 | M | Jun-99 | Narbonne | 3 | no |
| Languedoc-Roussillon | Gard (30) | 46 | 9 | M | Oct-98 | Bagnols-sur-Ceze | 3 | no |
| Languedoc-Roussillon | Hérault (34) | 47 | 47 | H | May-00 | Montpellier | 3 | no |
| Languedoc-Roussillon | Lozère (48) | 48 | 4 | M | Aug-96 | nm | 3 | no |
| Midi-Pyrénées | Aveyron (12) | 49  50  51 | 5 | F | Jul-04 | Millau | 2 | yes |
| Midi-Pyrénées | Aveyron (12) |  | 56 | M | Jul-98 | Millau | 2 | yes |
| Midi-Pyrénées | Aveyron (12) |  | 62 | M | May-99 | Sainte-Afrique | 2 | yes |
| Midi-Pyrénées | Aveyron (12) | 52 | 56 | F | Jun-99 | Millau | 3 | no |
| PACA | Alpes-de-Haute-Provence (04) | 53 | 4 | M | Aug-98 | Digne-les-Bains | 1 | no |
| PACA | Alpes-Maritimes (06) | 54 | 42 | H | Jul-99 | Nice | 1 | no |
| PACA | Alpes-Maritimes (06) | 55 | 30 | H | Sep-00 | Antibes | 1 | no |
| PACA | Alpes-Maritimes (06) | 56 | 41 | H | Jun-02 | Menton | 1 | no |
| PACA | Alpes-Maritimes (06) | 57 | 50 | M | Sep-02 | Nice | 1 | no |
| PACA | Var (83) | 58 | 15 | M | Jun-99 | Draguignan | 1 | no |
| PACA | Vaucluse (84) | 59 | 41 | M | Jul-99 | Valreas | 1 | no |
| PACA | Alpes-de-Haute-Provence (04) | 60 | 48 | F | Jul-95 | La Foux-d'Allos | 2 | yes |
| PACA | Alpes-de-Haute-Provence (04) | 61 | 62 | M | Jul-99 | Colmars-les-Alpes | 2 | yes |
| PACA | Alpes-de-Haute-Provence (04) | 62 | 70 | F | Aug-97 | Jausiers | 2 | no |
| PACA | Alpes-Maritimes (06) | 63 | 7 | M | Oct-02 | Grasse | 2 | no |
| PACA | Alpes-Maritimes (06) | 64 | 30 | M | May-02 | Menton | 2 | no |
| PACA | Alpes-Maritimes (06) | 65 | 32 | M | Oct-92 | Levens | 2 | yes |
| PACA | Alpes-Maritimes (06) | 66 | 40 | M | Aug-01 | Casterino | 2 | yes |
| PACA | Alpes-Maritimes (06) | 67 | 40 | M | Mar-02 | Menton | 2 | no |
| PACA | Alpes-Maritimes (06) | 68 | 45 | M | May-95 | Sospel | 2 | yes |
| PACA | Alpes-Maritimes (06) | 69 | 45 | F | Aug-05 | Vence | 2 | yes |
| PACA | Alpes-Maritimes (06) | 70 | 58 | M | Oct-00 | Levens | 2 | yes |
| PACA | Alpes-Maritimes (06) | 71 | 63 | M | Apr-93 | St-Martin-de-Vésubie | 2 | yes |
| PACA | Alpes-Maritimes (06) | 72 | 74 | F | Mar-97 | Peymeinade | 2 | no |
| PACA | Hautes-Alpes (05) | 73 | 7 | M | Jun-99 | Briançon | 2 | no |
| PACA | Var (83) | 74 | 51 | M | Sep-02 | Draguignan | 2 | no |
| PACA | Vaucluse (84) | 75 | 29 | M | May-99 | nm | 2 | no |
| PACA | Vaucluse (84) | 76 | 48 | M | Sep-99 | Pertuis | 2 | no |
| PACA | Alpes-de-Haute-Provence (04) | 77 | 27 | M | Jul-97 | Digne-les-Bains | 3 | no |
| PACA | Alpes-de-Haute-Provence (04) | 78 | 27 | M | Mar-98 | Sisteron | 3 | no |
| PACA | Alpes-de-Haute-Provence (04) | 79 | 88 | M | Sep-98 | Seynes | 3 | yes |
| PACA | Alpes-Maritimes (06) | 80 | 68 | F | Apr-97 | Auron | 3 | yes |
| PACA | Var (83) | 81 | 74 | F | Jul-98 | Brignoles | 3 | no |
| PACA | Vaucluse (84) | 82 | 45 | M | May-97 | nm | 3 | no |
| Pays-de-la-Loire | Loire-Atlantique (44) | 83 | 41 | M | Jul-00 | nm | 2 | no |
| Pays-de-la-Loire | Maine-et-Loire (49) | 84 | 2.6 | M | Sep-01 | nm | 2 | no |
| Pays-de-la-Loire | Maine-et-Loire (49) | 85 | 16 | M | Jul-99 | nm | 2 | no |
| Pays-de-la-Loire | Maine-et-Loire (49) | 86 | 29 | M | May-00 | nm | 2 | no |
| Pays-de-la-Loire | Maine-et-Loire (49) | 87 | 48 | M | Jun-00 | nm | 2 | no |
| Pays-de-la-Loire | Sarthe (72) | 88 | 19 | M | May-00 | nm | 2 | no |
| Pays-de-la-Loire | Sarthe (72) | 89 | 44 | M | Apr-01 | nm | 2 | no |
| Pays-de-la-Loire | Vendée (85) | 90 | 18 | F | Jul-00 | nm | 2 | no |
| Rhône-Alpes | Isère (38) | 91 | 36 | M | Jul-02 | Septème | 1 | no |
| Rhone-Alpes | Drôme (26) | 92 | 40 | M | Mar-97 | nm | 3 | no |
| Rhone-Alpes | Isère (38) | 93 | 38 | F | Aug-96 | nm | 2 | no |
| Rhone-Alpes | Isère (38) | 94 | 40 | M | Jun-00 | Grenoble | 2 | no |
| Rhone-Alpes | Isère (38) | 95 | 62 | M | Aug-00 | Septème | 2 | no |
| Rhone-Alpes | Isère (38) | 96 | 4 | M | Apr-96 | Romans-sur-Isère | 3 | no |
| Rhone-Alpes | Drôme (26) | 97 | 50 | M | Sep-97 | Bourg-de-Péage | 3 | no |

Epidemiological data from 97 patients selected from 110 cases of envenomations collated from 1990 to 2005 in France. The gradation was established according to [24]. PACA: Provence-Alpes-Côte-d’Azur.
